# Curcumin: Biological, Pharmaceutical, Nutraceutical, and Analytical Aspects

**DOI:** 10.3390/molecules24162930

**Published:** 2019-08-13

**Authors:** Raghavendhar R. Kotha, Devanand L. Luthria

**Affiliations:** USDA-ARS, Beltsville Human Nutrition Research Center, Beltsville, MD 20705, USA

**Keywords:** curcumin, biological activity, formulations, sample preparation, analytical methods

## Abstract

Turmeric is a curry spice that originated from India, which has attracted great interest in recent decades because it contains bioactive curcuminoids (curcumin, demethoxycurcumin, and bisdemethoxycurcumin). Curcumin (1,7-bis-(4-hydroxy-3-methoxyphenyl)-hepta-1,6-diene-3,5-dione), a lipophilic polyphenol may work as an anticancer, antibiotic, anti-inflammatory, and anti-aging agent as suggested by several in vitro, in vivo studies and clinical trials. However, poor aqueous solubility, bioavailability, and pharmacokinetic profiles limit curcumin’s therapeutic usage. To address these issues, several curcumin formulations have been developed. However, suboptimal sample preparation and analysis methodologies often hamper the accurate evaluation of bioactivities and their clinical efficacy. This review summarizes recent research on biological, pharmaceutical, and analytical aspects of the curcumin. Various formulation techniques and corresponding clinical trials and in vivo outcomes are discussed. A detailed comparison of different sample preparation (ultrasonic, pressurized liquid extraction, microwave, reflux) and analytical (FT-IR, FT-NIR, FT-Raman, UV, NMR, HPTLC, HPLC, and LC-MS/MS) methodologies used for the extraction and quantification of curcuminoids in different matrices, is presented. Application of optimal sample preparation, chromatographic separation, and detection methodologies will significantly improve the assessment of different formulations and biological activities of curcuminoids.

## 1. Introduction

The use of nutraceuticals, dietary supplements, and functional foods has gained significant popularity globally over the past few decades due to increased interest in natural products and their potential health benefits [1,2,3,4]. In addition, natural products are often perceived as less toxic compared to synthetically derived products [5]. Turmeric, a plant-derived curry spice, has been recognized for its medicinal properties [2]. Due to these advantages, several turmeric dietary supplements are available in the global market with varying formulations and health claims (supporting joint comfort, promoting mobility and flexibility, enhancing cognitive functioning, and providing cardiovascular health benefits) [1,2,3,4,5].

Turmeric (*Curcuma longa*) is a plant related to the ginger family (*Zingiberaceae*), which originated from India and is currently grown in several other parts of the world, including Southeast Asia, China, and Latin America [6,7]. Turmeric is a common spice used in the preparation of curries in India and other Asian countries because of its flavor and color [2,8]. India is the largest producer [9] and leading turmeric exporter. As per reports, the global market for turmeric was estimated at ~1.7 million metric tons and was expected to increase significantly by 2027 [10,11]. The curcumin global market was estimated to be around half a billion US dollars in 2016 and is projected to register a compound annual growth rate (CAGR) of 13% over the period of 2018–2025 [10].

Apart from its use as a curry spice, turmeric has been historically used as a natural coloring agent (food, cosmetics, and textiles), an insect repellent, and as an antimicrobial agent [12]. As per Ayurvedic medicine, turmeric has been used for various medical purposes such as wound healing, respiratory problems, liver, and dermatological disorders [5,8,13]. Curcumin (CUR), demethoxycurcumin (DMC), and bisdemethoxycurcumin (BMC) are bioactive polyphenolic compounds identified in turmeric [14,15], which have been collectively referred to as curcuminoids (CCMs). As per the United States Food and Drug Administration (FDA), curcuminoids are generally recognized as safe (GRAS) [15]. Clinical studies further supported that a high single oral dose (up to 12 g/day) of curcuminoids were very well tolerated [16,17]. 

**Figure 1:**
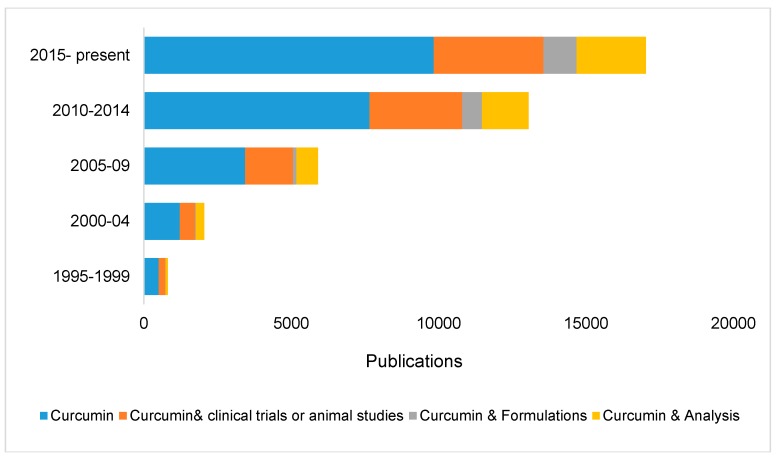

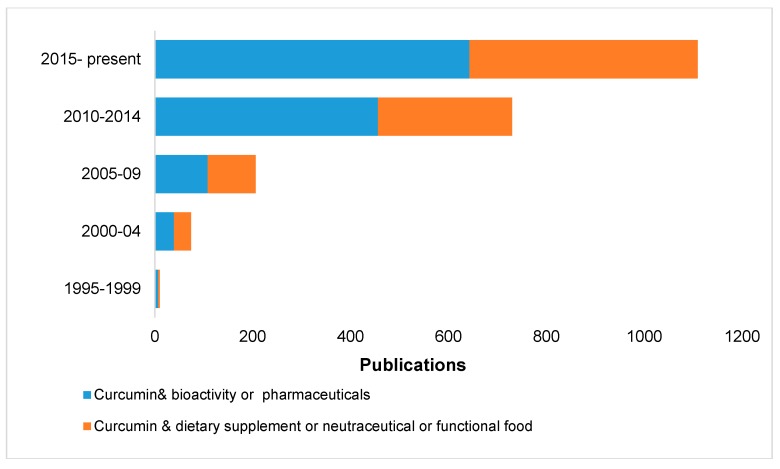
(**Top**) The number of publications published since 1995 on (i) curcumin, (ii) curcumin and clinical trials or animal studies, (iii) curcumin and formulations, and (iv) curcumin and analysis. (**Bottom**) (v) curcumin and bioactivity or pharmaceuticals, and (vi) curcumin and dietary supplement or nutraceutical or functional food.

Curcumin has attracted a lot of attention in recent decades due to its therapeutic potential as anti-inflammatory, anti-diabetic, anti-cancer, and anti-aging agent [5,8,15,18], which is supported by several in vitro, in vivo and clinical trials [6,14,19,20,21]. In addition to these, curcumin has also shown promise in treating wound healing, arthritis, and Alzheimer’s. Figure 1 shows that significant increase in curcumin-based research. Based on Scopus research, almost 10,000 research articles have been published on curcuminoids in the past five years. However, curcumin therapeutic potential is limited by its low solubility in aqueous media, poor bioavailability, and pharmacokinetic profiles [17,18,22,23,24]. To address these issues, several different formulations (materials/ mixtures that combined curcumin with other elements, including polymers, lipids, and nanoparticles in appropriate proportions) have been produced and used in multiple studies [6,14,19,20,21]. 

Different sample preparation and analytical techniques have been developed to analyze CCMs [25,26], however, often detailed optimization information is not presented. It is essential to understand how different sample preparation and analysis techniques impact the quantification, bioavailability, pharmacokinetic profiles, and clinical efficacy of CCMs [25,26,27,28,29,30,31,32,33], which in turn will assist in increasing understanding of curcuminoids as a potential therapeutic agent [7,26,31,34]. This review presents current research updates in the areas of CCMs formulations, and their clinical outcomes [6,14,19,20,21], sample preparation [25,26,27,28,29,31,32,33,35,36,37,38,39], and various methodologies used to analyze CCMs [25,26,33,38,39,40,41,42,43,44,45,46,47,48,49]. Sample preparation techniques developed for extracting CCMs from plants, dietary supplements, and other biological matrices, have been described. Applications of different spectroscopy and chromatography methodologies used for detection of adulterants, identification, and quantification of CCMs from various matrices has also been discussed. Details of extraction conditions, efficiency, limits of quantification, and detection using liquid chromatography coupled with tandem mass spectrometry (LC-MS/MS) have been tabulated. Furthermore, we have discussed the advantages and limitations of different analytical techniques with the emphasis on sensitivity, accuracy, and robustness.

## 2. Curcumin

### 2.1. Chemistry

**Figure 2:**
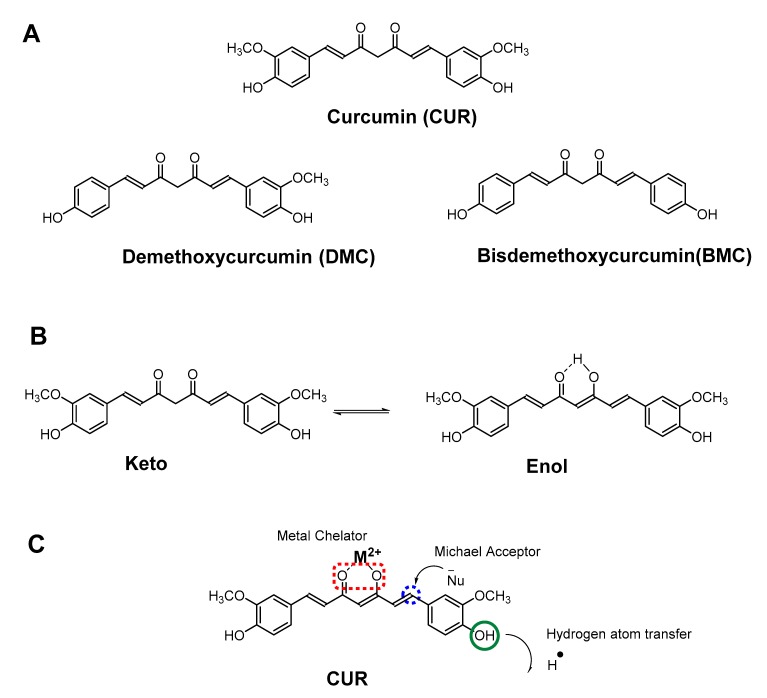
(**A**). Chemical structures of curcuminoids, (**B**). Keto-enol tautomers of curcumin, and (**C**). Chemical reactivity sites in curcumin which contribute to its activity and bioavailability.

Chemical composition of turmeric consists of approximately 70% carbohydrates, 13% moisture, 6% protein, 6% essential oils (phellandrene, sabinene, cineol, borneol, zingiberene and sesquiterpenes), 5% fat, 3% mineral (potassium, calcium, phosphorus, iron, and sodium), 3–5% curcuminoids, and trace amounts of vitamins (B1, B2, C, and niacin) [5,17,24,50,51]. Among the curcuminoids (Figure 2A), approximately CUR accounts for 77%, DMC accounts for 17% and BMC accounts for 3–6% [17,18].

Curcumin was first isolated by Vogel and Pelletier in 1815 from the rhizomes of *C. longa* [17,52]. In 1842, Vogel Jr purified curcumin for the first time [17]. After several decades, in 1910, Melabedzka et al. reported the structure of curcumin as diferuloylmethane, or 1,6-heptadiene-3,5-dione-1,7-bis (4-hydroxy-3-methoxyphenyl)-(1*E*,6*E*) (Figure 2A) [17]. Three years later, in 1913, Lampe and Melobedzka reported a method for synthesizing curcumin [17,50,53]. In 1953, Srinivasan reported separation and quantification of curcumin components using chromatography [17,54]. 

Curcumin has two tautomeric forms, keto- and enol (Figure 2B). Curcumin is practically insoluble at room temperature in aqueous solutions at neutral and acidic pH. However, due to lipophilic nature with a log *P* value of ~3.0, it is soluble in organic solvents like methanol, ethanol, acetone, and dimethyl sulfoxide. Both at neutral and acidic pH, keto form is predominant, however, enol tautomer is exclusively present in alkaline conditions, which can be rationalized by the intramolecular hydrogen bonding in enol form [50,55,56]. The solubility of curcumin in aqueous solution increases under alkaline conditions, but CUR degrades rapidly under both neutral and alkaline conditions. Curcumin shows maximum absorption at 430 nm in methanol and 415–420 nm in acetone [5,57]. In alkaline conditions (pH > 10), CUR is fully deprotonated and shows maximum absorbance at 467 nm. Curcumin has pKa of 8.54 and possesses three labile protons at neutral pH of which one is enolic, and two are phenolic protons. 

CUR primarily has three reactive sites, as illustrated in Figure 2C, hydrogen atom donor, Michael acceptor, and a metal chelator [23,24,50]. The α,β-unsaturated β-diketone portion of CUR is an excellent metal chelating agent and forms complexes with several known metal ions. The metal chelating ability of CUR has shown great promise as a therapeutic agent against diseases like Alzheimer’s, cancer, depression, and arthritis [50,58,59,60,61]. CUR forms complexes with all the metals such as Al^3+^, which are involved in Alzheimer’s, or direct binding to the small β-amyloid species to prevent aggregation and fibril formation [62,63,64,65,66]. Curcumin reduces heavy metal-induced toxicity (oxidative stress) by forming stable complexes with heavy metals like copper (Cu), chromium (Cr), arsenic (As), mercury (Hg), lead (Pb) and cadmium (Cd) [67,68,69,70,71,72]. The β-diketo moiety of curcumin not only reacts as a metal chelator but also involves as a Michael acceptor in nucleophilic addition reactions which occur between a nucleophile (like -OH, SH- and -SeH) and the α,β-unsaturated ketone moiety of curcumin (Figure 2C). In particular, thiol (-SH) groups in glutathiones and enzymes containing -SH and selenol (-SeH) groups, have been shown to react with α,β-unsaturated ketone moiety of curcumin [50,73,74,75,76,77].

### 2.2. Bioactivity

CUR has an ability to affect multiple biological targets and has been shown to exhibit activity against various diseases, including cancer, cardiovascular disease, neurological and autoimmune diseases) [17,18,19,54]. CUR can modulate several biological targets (including transcription factors, growth factors, inflammatory mediators, cytokines, cell cycle proteins, enzymes, protein kinases, and apoptotic proteins) and cellular pathways [7,17,78]. For instance, CUR modulates tumor growth via regulating multiple signaling pathways, including cell survival, tumor suppressor, caspase pathways, protein kinase, and death receptor pathways [5,7,19]. 

CUR can inhibit the activation of the transcription factor nuclear factor kappa B (NF-_K_B), which is responsible for cell survival, cytokine production, and other cellular functions. CUR downregulates the signal transducer and activation transcription (STAT) proteins, which are essential for cell growth, differentiation, and survival [5,19,23]. STAT proteins are also involved in immune system development, function, and clearance. Curcumin upregulates a leucine zipper protein NrF2, which regulates the expression of antioxidant properties that protect the cells from oxidative stress [5,19,23]. Curcuminoids therapeutic effect in treating some of the medical conditions/diseases and related mode of action have been discussed below.

**Cancer:** Garcea et al. reported that CUR administration resulted in the reduction of M(1)G protein levels, while COX-2 protein levels were unchanged in a malignant colorectal tissue [79]. He et al. reported curcumin treatment overexpressed P53 levels in tumor cells of colorectal cancer patients, which in turn promoted apoptosis [80]. Kim et al. studied the effect of curcumin as an anti-inflammatory agent in HNSCC (head and neck squamous cell carcinoma cancer) patients [81]. In this study, curcumin was found to suppress inflammatory cytokines such as TNF-α, IKKβ kinase, IL-6, and IL-8. CUR can also suppress the activity of protein kinases, including protein kinase (PK)A, phosphorylase kinase (PhK), the mammalian target of rapamycin (mTOR), and mitogen-activated protein kinases (MAPKs) [23] which play essential roles in various cellular responses, including regulation of cell growth, proliferation, division, survival and death.

**Cardiovascular:** Sahebkar reported that curcuminoids might reduce circulatory C-reactive protein levels, which is a predictor and independent risk factor of cardiovascular disease [82]. Curcumin has also shown to be effective against myocardial infarction and atherosclerosis [83]. CUR also has shown to decrease triglycerides levels, LDL (low-density lipoprotein), and total cholesterol [19]. In another study by Swamy et al. curcumin prevented doxorubicin-induced cardiomyopathy [84]. It increased the level of glutathione (GSH), superoxide dismutase (SOD), and catalase (CAT), while decreased the elevated malondialdehyde (MDA) levels.

**Diabetes:** CUR was found to be effective in treating diabetes in patients and animal models. Arun and Nalini reported that turmeric or curcumin administration to diabetic rats reduced sugar and hemoglobin levels in the blood [85]. Murugan and Pari in 2007 observed that decreased levels of plasma total protein, albumin, globulin, and albumin/globulin ratio in diabetic rats were brought back to near normal after CUR administration [86]. In diabetic rat models, oral administration of CUR resulted in a significant reduction in blood glucose and a significant increase in plasma insulin levels [86]. 

While a large number of in vivo experiments and clinical trials claim that CUR has great potential as a therapeutic agent, additional research is needed to confirm and validate the beneficial health claims of CCMs [24]. Pure CUR has been used in many in vitro studies, whereas most in vivo studies and clinical trials use CCMs mixture [24]. These terms (CUR and CCMs) have often been used as synonyms in literature [7,24]. Pagano et al. recently published an overview on the systemic reviews related to the efficacy of curcumin containing nutraceuticals for different health issues [87]. The authors established the search strategy using ’curcum’ or ‘turmeric’ and ‘systematic review’ or ‘meta-analysis’ or ‘clinical trials.’ The authors evaluated the methodological and reporting quality of previous studies using A measurement tool to assess systematic reviews (AMSTAR) and Overview quality assessment questionnaire (OQAQ). The results showed that only 22 systematic reviews (SR) met the authors’ inclusion criteria. Only four of the SRs scored high on the AMSTAR scale, and 12 SRs were high on OQAQ scores. The authors concluded that the efficacy of curcumin containing nutraceuticals was well demonstrated for several health conditions, including skin diseases, arthritis-related diseases, metabolic diseases, and depressive disorders. However, due to the poor quality of primary trials (i.e., number of trials for each systematic review, design, participants, and duration of the treatment) and the analysis methodology used, the uncertainty in bioefficacy of curcumin still exists [87]. 

### 2.3. Bioavailability

The therapeutic potential of CUR is mainly circumvented by its low bioavailability and poor pharmacokinetic profile (ADME; absorption, distribution, metabolism, and excretion) and short half-life time in the gastrointestinal (GI) tract [18,23]. Another challenge for CUR as a potential therapeutic agent is its poor stability under physiological conditions. For instance, at 37 °C and neutral pH (7.2), curcumin *t_1/2_*was reported less than 10 min [24]. CUR degradation occurs in two pathways: solvolysis and photodegradation [24]. Solvolysis involves the nucleophilic substitution or elimination by solvent molecules. The nucleophile attack occurs on α,β-unsaturated ketone part of CUR (Michael addition). In aqueous alkaline buffer, solvolysis of heptadienone chain results in 90% of CUR degradation to generate vanillin, ferulic acid, ferulic aldehyde, and other products [18,24,50]. Autoxidation of CUR occurs via a radical chain reaction that leads to oxygen incorporation to yield a bicyclopentadione product [22,24]. Several reports suggested that CUR is sensitive to light both in solid and solution forms; hence, curcumin samples should be protected from light exposure. CUR degradation occurs upon exposure to sunlight [50,55] which is commonly observed by quick removal of turmeric stains when exposed to sunlight. Photochemical degradation of CUR occurs in solid as well as solubilized forms to ferulic acid, ferulic aldehyde, vanillin, and vanillic acid [24,50].

**Figure 3:**
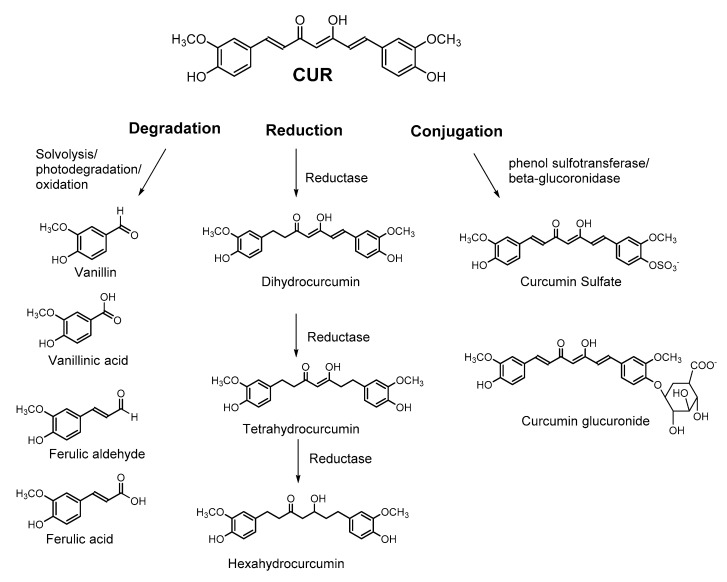
Curcumin degradation and metabolism products.

CUR metabolism mainly occurs via reduction and conjugation [17,18,22,50,88,89,90]. CUR degradation and metabolism products are shown in Figure 3 [17,18,22,50,88,89,90]. Reduction of CUR primarily occurs through the heptadienone chain double bonds to form di-, tetra-, and octahydrocurcumin, which is facilitated by several enzymes such as NADPH-dependent reductase, alcohol dehydrogenase and an unidentified microsomal enzyme [18,22,50]. Glucuronidation/sulfonation conjugation is the other primary metabolic pathway of CUR in the body. Conjugation primarily occurs at the phenolic oxygen of curcumin via enzymatic reactions [50]. For instance, O-sulfonation of CUR is catalyzed by human phenol sulfotransferase isoenzymes SULT1A1 and SULT1A3 [17,88]. Similarly, glucuronidation is catalyzed by UDP-glucuronosyltransferase [89].

## 3. Formulations

Various types of chemical modifications of CUR (which include use of liposomes, nanoparticles, micelles, phospholipid complexes, polymers, adjuvants) have been developed to improve curcumin solubility, bioavailability, longer circulation time, targeted delivery and ADME profiles [6,18,19,20,21,90,91,92,93,94,95,96,97,98,99,100]. Nano-, micro-formulations have gained a great focus because of the advantages associated with them, including increased solubility, improved cellular uptake, target specificity), decreased degradation, increased bioavailability, circulation times, and ADME profiles [7,101]. Various types of CUR formulations and delivery systems have been reported in the literature, including liposomes, lipid-based nanoparticles, polymeric nanoparticles, micelles, microemulsions, metal-based nanoparticles [21]. Table 1 summarizes some of the CUR formulations used for animal studies and clinical trials along with their outcomes.

Among the multiple curcumin-formulations [15,21,102,103,104,105,106,107,108,109,110,111,112,113,114,115,116,117,118,119,120,121,122,123,124,125,126,127,128,129], the phytosomal formulation of CUR (Meriva), which is a complex of curcumin with phosphatidylcholine, is one of the well-studied curcumin-formulation (Table 1). Meriva is prepared by adding phospholipids to the hydroalcoholic extract of turmeric rhizomes under reflux conditions [21]. Meriva has shown improved bioavailability and pharmacokinetic profiles than the uncomplexed curcumin [21]. There have been several studies focusing on the efficacy of phytosomal curcumin in treating conditions such as cancer, inflammatory diseases, and diabetes. The data suggest that the phytosomal curcumin formulation has excellent properties as a delivery system [15,21].

Belcaro et al. reported a clinical study to assess a proprietary lecithin delivery system of CUR (Meriva) during chemo- and radiotherapy in 160 cancer patients [112]. The authors concluded that formulated CUR could potentially lower the pain side-effects caused during cancer therapy. In another study, a randomized, double-blind, placebo-controlled trial was carried out by Panahi et al. [103] on 80 subjects with solid tumors. This study supported the clinical efficacy of curcuminoids adjuvant therapy by improving the quality of life (QoL) of patients with solid tumors. In another study, an eight-week open, random, controlled clinical trial was conducted on 141 patients who were affected by neuropathic pain [113]. The purpose of the study was to evaluate the role of adjunctive therapy (a multi-ingredient formulation which consists of lipoic acid, CUR, and piperine) to dexibuprofen administration. This treatment indeed reduced neuropathic pain by more than 2/3. Moreover, this treatment reduced dexibuprofen usage by 40% [113].

Theracurmin, another CUR formulation consisting of dispersed curcumin with colloidal nanoparticles, was studied for improved bioavailability [114,115] and its efficacy in treating osteoarthritis compared to turmeric powder itself [116]. Theracurmin was shown to have greater bioavailability than turmeric powder by 40 fold in rats and 27 fold higher in humans [114]. A clinical study conducted by Nakagawa et al. concluded that Theracurmin was found to be effective in decreasing pain without causing any side effects [116].

Apart from Meriva and Theracurmin, several other CUR formulations have been reported in the literature [117,118,119,120,121,122,123,124,125,126,127,128,129]. Kakkar and Kaur reported a significant reduction in AlCl_3_ related neurotoxicity in male lacca mice [117] using a solid lipid nanoparticle CUR formulation [117]. Similarly, Nayak et al. reported curcuminoids loaded nanoparticles, which showed improved antimalarial activity in Albino mice [121]. In a clinical study on 60 periodontitis patients treated with CUR collagen sponge, Gottumukkala et al. found a significant reduction in the clinical (plaque and gingival index scores) and microbiological (BANA test and microbial colony count) parameters [129].

## 4. Sample Preparation for Analysis

Sample preparation plays a vital role in the accurate quantification of analytes from various matrices [7,34]. Often the concentration of the analyte of interest in samples is low and endogenous matrix components, and their degraded products (generated at elevated temperatures during the sample preparation) may interfere with the assay of the analyte of interest [27,28]. As a wide range of formulations for curcuminoids has emerged in the global market, there is a distinct need for developing robust, validated methods for their extraction and analysis [7]. This will also be important for the analysis of their biosafety, toxicity, bioactivities, and other health claims. Hence, an ideal sample preparation method should be simple, fast, and yielding maximum analyte recovery efficiently and minimum (or no) matrix interferences. Table 2 shows various sample preparation protocols used for extraction and analysis of curcuminoids from a wide array of matrices (biological-plasma and tissue, plant -rhizome and foods) as well as different formulations [13,25,26,27,28,29,31,32,33,35,36,37,38,39,130,131].

CUR extraction from powdered rhizomes has been carried out using various extraction techniques, conditions, and solvents (Table 2). Among those methods, ultrasonication, reflux, pressurized liquid extraction, and microwave-assisted extractions are commonly used [13,29,32,36,38]. Liu et al. carried out CUR extraction from powdered rhizome using ultrasonication with acetonitrile: ethanol mixture (1:2, *v*/*v*) with 82% recovery efficiency [38]. In another case, Ashraf et al. reported CUR extraction using reflux in methanol for two hours at 70 °C with 99% recovery [29]. Similarly, Chao used pressurized liquid extraction technique (100 °C temperature and 1500 psi of pressure) with ethanol as a solvent to quantitatively (93–101%)recover curcumin [32]. Dandekar and Gaikar reported a microwave-assisted extraction (MAE) of curcuminoids from turmeric into various organic solvents such as acetone, ethyl alcohol, and isopropyl alcohol [132]. Using their optimized method, they extracted 60% of curcuminoids in acetone in 1 min. Later, Wakte et al. reported that water-soaked irradiated Curcuma powder in acetone solvent yielded 90% recovery of curcuminoids in 5 min [13].

Sample preparation techniques to extract CUR from different formulations and matrices are shown in Table 2. Kumar et al. used a simple sonication method using methanol solvent to extract ~99% of curcumin from a tablet form [33]. Similarly, Liang et al. used an extraction method with methanol and diluted HCl to extract CUR quantitatively (93–101%) from Tiantai No.1 pills [31]. Silva-Buzanello et al. reported curcumin extraction from poly (L-lactic acid) nanoparticles by adding dichloromethane to dried nanoparticles and precipitated polymer by adding methanol [39]. Using this method, authors reported ~86% of recovery of curcumin [39].

Protein precipitation and liquid-liquid extractions are commonly used to extract analytes (drugs and their metabolites) from biological samples (plasma and tissues). Kunati et al. reported CUR extraction from human plasma using protein precipitation in methanol with 0.1% formic acid [25]. After protein precipitation, the resulting solution was centrifuged, the supernatant was collected, dried under nitrogen and reconstituted in an appropriate solvent for analysis. Similarly, Li et al. used acetonitrile protein precipitation, followed by size-exclusion chromatography [26]. After the protein precipitation, the resulting mixture was centrifuged, and the supernatant was transferred to an OMEGA NANOSEP 10K nanosep size-exclusion tube. The resulting extract was used for the analysis. Jade et al. extracted curcumin from human plasma using ethyl acetate (liquid-liquid extraction) for three times. Ramalingam analyzed curcumin from mouse plasma and brain tissue by adding sodium hydroxide followed by ethyl acetate extraction [28].

From the literature summary presented in Table 2, Table 3 and Table 4, it is evident that the optimization of the sample preparation has not been the focus of peer-reviewed publications. This is evident as a wide range of solvents, solid-to-solvent ratio, extraction times, and techniques used for the analysis of the curcuminoids from different matrices. Recently, Yulianto et al. [130] and Chao et al. [32] used a pressurized liquid extractor for the extraction of curcuminoids from the powdered rhizome. Chao determined that the optimum conditions for the extraction of curcuminoids on the laboratory scale using accelerated solvent extractor was achieved with ethanol as extraction solvent at 100 °C, 1500 psi pressure, extraction time of 5 min, and a flush volume of 60% [32]. However, when water was used as an extraction solvent, the temperature was the critical factor that impacted the extraction efficiency [130]. Therefore, optimization of extraction and analysis protocols are needed to quantify curcuminoids content in different matrices accurately.

## 5. Methods for Qualitative and Quantitative Analysis

Sensitive, accurate, and robust analytical methods are required for CUR quantification because a) CUR levels in plasma/serum/tissues are low due to poor bioavailability, rapid metabolism, and degradation, b) matrix interferences, and c) proper evaluation of bioefficacy and understanding mechanism/mode of action [7,26].

Each analytical technique has its advantages and limitations. For instance, thin layer chromatography (TLC) is an analytical technique classically used to separate analytes from mixtures/matrix interferences. This technique is relatively easy to operate; however, it provides poor resolution and is primarily useful for the qualitative/semi-quantitative analysis of analytes that occur in comparatively higher concentrations. On the other hand, LC-MS/MS is a powerful analytical tool for the sample analysis, both qualitatively and quantitively. Tandem mass spectrometry techniques provide not only accurate structural information of the analyte but also accurate measurements of the analytes even at very low concentrations (nano to picogram/mL) [7,26,133]. On the downside, LC-MS/MS instrumentation is expensive and requires a significant amount of technical expertise and maintenance. The techniques used for sample analysis are primarily dependent on the instrument availability and the research objective of the study. Table 3 [33,38,39,40,41,42,43,44,45,46,47,48,49,134,135] and Table 4 [14,25,26,27,28,29,30,31,35,36,37,93,96,133,136,137,138,139,140,141,142,143,144,145] summarize the various instrumentation and methods used to study and quantify curcumin in different matrices.

Spectroscopy techniques like Fourier transform (FT) infrared (IR), near-infrared (NIR), Raman, and ultraviolet/visible (UV-Vis) spectroscopic methods have been used for qualitative and quantitative analysis of curcuminoids and commonly found adulterants. Dhakal et al. used FT-IR and Raman for easy identification of the adulterant (metanil yellow and Sudan-I) from ground turmeric. The authors concluded that FT-Raman is more sensitive than FT-IR in detecting metanil yellow in turmeric [41]. Tanaka et al. used NIR coupled with multivariate analysis to quantify curcuminoids in Curcuma rhizome and compared results with HPLC quantification [43]. Authors observed that prediction by partial least-squares regression showed high correlation with HPLC quantification results. Curcuminoids show strong UV-Vis absorption at a λ_max_ of 425 nm [24,50] and UV-Vis spectroscopy can easily be used for quantification of CCMs if sample matrix or other components present have no absorption in this range. The main advantages of spectroscopic based analysis are speed, ease, and cost-effectiveness. However, the above techniques are not as sensitive as mass spectrometry and often suffers from the matrix interferences that have components showing absorption at the same wavelength as the analyte. Spectroscopic methods (UV, IR, and NIR) can be used as a high throughput screening tools. All the spectroscopic techniques are influenced by matrix and often require confirmation using more specific detection techniques (HPLC or HPLC coupled with mass spectrometry) [7].

Chromatography is a powerful and robust technique for both qualitative and quantitative analysis of various types of analytes [7,34]. In particular, more than 2/3 of the analytical methodologies for CUR quantification are based on separation techniques with HPLC being the most dominant choice [7]. Chromatography is a separation technique, where analytes are separated based on their physical and chemical properties. Thin layer chromatography (TLC) and high-performance thin layer chromatography (HPTLC) are the separation techniques which address the issue of matrix interferences. While TLC primarily is used for qualitative analysis purpose, HPTLC can be used for both qualitative and quantitative purpose. The disadvantage of the HPTLC technique is a lack of sensitivity, resolution, and issues associated with the reproducibility. Table 3 summarizes the details of different non-LC-MS methods used in the literature for detecting adulteration and analysis of curcuminoids for different matrices.

HPLC techniques have been extensively used for the quantification of analytes in various fields. HPLC can be coupled to different detectors such as UV, fluorescence, and mass spectrometer (MS) (Table 3 and Table 4). While all detectors have their own advantages and limitations, MS has been proved to be a very useful, sensitive, accurate, and robust detector. For the separation of CUR using HPLC, mostly reverse-phase liquid chromatography, particularly C18 columns with different particle sizes have been used. While most of the reports are based on C18 columns, C8 and other columns have been used for CUR separation (Table 4). In recent years, due to the surge in animal studies and clinical trials on CUR efficacy as a therapeutic agent, there has been a high demand for rapid and sensitive analytical methods to quantify curcumin in trace quantities. As a result, a number of LC-MS/MS based methods have been developed and validated as MS detectors offer greater sensitivity and accuracy in quantification, as presented in Table 4 [14,25,26,27,28,29,30,31,35,36,37,93,96,133,136,137,138,139,140,141,142,143,144,145]. For instance, Huang et al. recently reported a validated LC-MS/MS method to quantify CUR and other compounds with an excellent low limit of quantification (LLOQ) of 0.01 ng/mL (10 pg/mL) and low limit of detection (LOD) of 0.004 ng/mL (4 pg/mL) [133]. Similarly, Ahmad et al. reported a validated LC-MS/MS method with LLOQ of 0.05 ng/mL and LOD of 0.02 ng/mL for curcumin quantification in rat brain homogenate and plasma [139].

## 6. Conclusions

The evidence for curcumin (CUR) as a potential therapeutic agent and nutraceutical has increased in recent decades. This is evident from the development of a large number of curcumin formulations. This increased interest has spurred the growth of in vivo, in vitro, clinical trials to evaluate the bioefficacy of CUR and curcuminoids. To support this research, there have been developments in sample preparation and analyses methodologies to screen, isolate, and quantify curcuminoids from different matrices and detect adulterants. Accurate analysis of curcuminoids in various plant matrices, formulations, and biological samples, has become an important aspect to evaluate CCMs efficacy, bioavailability, and pharmacokinetic profiles accurately.

Extraction of curcuminoids from plant, food and formulations matrices can be accomplished using an alcohol solvent (methanol or ethanol) with either ultrasonication, reflux or pressurized liquid extraction techniques. For extracting curcuminoids from in vitro, in vivo, and clinical samples, protein precipitation using acetonitrile/methanol and liquid-liquid extraction techniques are preferred. Spectroscopic methods can be used for rapid screening of curcuminoids and detection of adulterants in turmeric and related products. For accurate quantification and detection of trace amounts of curcuminoids and metabolites, chromatographic separation coupled to mass spectrometry detection (LC-MS/MS) methods should be used as they provide high accuracy, reproducibility, and high sensitivity with low LOD and LLOQ.

## Figures and Tables

**Table 1:** Several curcumin formulations and their clinical or in-vivo outcomes.

Disease/Curcumin Activity	Formulations	Clinical Trial/In Vivo	Outcome	Ref
	Curcumin with a combination of hydrophilic carrier, cellulosic derivatives, and natural antioxidants	Randomized, double-blind, crossover human study in 12 healthy volunteers	Significantly increases curcuminoid appearance in the blood in comparison to unformulated standard curcumin.	[90]
Solid tumor	Meriva^®^ (Patented and commercialized); a complex of curcumin with phosphatidylcholine	Clinical trial (80 patients undergoing chemotherapy)	Suppression of systemic inflammation via reduction of inflammatory mediators and biomarkers (TNF-α, CGRP, substance P, MCP-1, hs-CRP, and IL-6)	[103]
Diabetes	Meriva^®^	38 patients	Significant improvement in the venoarteriolar response and a decrease in the peripheral oedema	[105]
Osteoarthritis	Meriva^®^	Clinical trial (100 patients)	Improvement of both the clinical and biochemical endpoints	[106]
Alzheimer’s	CUR loaded p(PEG-PLA) micelles	Tg2576 mice	Improved bioavailability in brain, and significant improvements in working and cue memory	[108]
Central serous chorioretinopathy	Meriva^®^	Clinical trial (12 patients)	Reduction in neuroretinal or retinal pigment epithelium detachment	[109]
Osteoarthritis	Meriva^®^	Clinical trial (50 patients)	Clinically effective in osteoarthritis treatment and management, while treatment costs were reduced significantly	[110]
Diabetes	Meriva^®^	25 patients	Decrease in skin flux and edema	[111]
Solid tumor	Meriva^®^	Clinical trial (160 patients undergoing chemo- and radiotherapy)	Signs of reduced side effects of cancer chemo- and radiotherapy treatment which are attributed to an anti-oxidant and anti-inflammatory activity of curcumin	[112]
Peripheral neuropathy	Lipicur (a mixture of lipoic acid, curcumin phytosome and piperine)	Clinical trial (135 patients)	Reduction in neuropathic pain and usage of the drug significantly.	[113]
	THERACURMIN (Nano-particle colloidal dispersion,)	Male Sprague-Dawley rats and 7 healthy human volunteers	Improved bioavailability than turmeric powder; 40-fold in rats and 27-fold in human	[114]
	THERACURMIN	6 healthy human volunteers	Improved bioavailability	[115]
Osteoarthritis	THERACURMIN	50 patients with knee osteoarthritis	Significantly effective in decreasing pain and NSAID necessity without any major side effects	[116]
Reversing neuronal damage	Solid lipid nanoparticles	In vivo (male Lacca mice)	Significantly reduced the AlCl3 related neurotoxicity	[117]
Cerebral ischemia	Solid lipid nanoparticles	Male Wistar rats	Showed significant effect against cerebral ischemia	[118]
Antiglioma activity	Curcumin-loaded lipid-core nanocapsules (C-LNCs)	Male Wistar rats	Decreased the tumor size and malignance and prolonged animal survival	[119]
Bioavailability	Curcumin micelles	Female NMRI mice	10–40- Folds increase in bioavailability in plasma and brain of mice	[120]
Antimalarial activity	Curcuminoids loaded lipid nanoparticles	Albino mice	Twofold improvement in antimalarial activity	[121]
Antimalarial activity	curcuminoids loaded liposomes	Albino mice	Showed lower parasitemia and higher survival than the control group	[122]
Periodontitis	Curcumin gel	25 patients	Significant reduction in periopathogens	[123]
Wound healing	Curcumin-loaded hyalurosomes	Female CD-1 mice	Reduced inflammation and injuries, diminishing oedema formation, and myeloperoxidase activity	[124]
Diabetic wound healing	CUR-CSNPs impregnation into collagen scaffold	Adult male Wistar rats	accelerated the cutaneous wound healing by decreasing the inflammation	[125]
Wound healing	Cur- polymer micelle loaded thermosensitive hydrogel	Male Sprague-Dawley (SD) albino rats	Enhanced the cutaneous wound healing process	[127]
Diabetic wound healing	Curcumin-loaded nanofibers	Adult male Sprague- Dawley rats	increasing the collagen content in treating diabetic wounds and effectively promotes healing of such wounds in the early stages	[128]
Periodontitis	curcumin collagen sponge	60 patients	Significant reduction in all the clinical and microbiological parameters	[129]

**Table 2:** Several sample preparation techniques used for the extraction of curcuminoids.

Matrix	Pretreatment/Extraction Approach	Procedure	Recovery %	Ref
**Biological Samples**				
Human plasma	Protein precipitation in methanol	10 μL of IS mix was added to plasma, vortex-mixed for 30 s. 800 μL of methanol with 0.1% formic acid was added for protein precipitation. The resulting solution was vortex-mixed, centrifuged and the supernatant was dried under nitrogen. The residue was reconstituted in methanol: ammonium formate (1:1).	72–84%	[25]
Rat plasma	Acetonitrile protein precipitation coupled with size-exclusion chromatography	100 μL of Plasma samples were spiked with CUR-d6 IS and mixed with 300 μL of cold acetonitrile and vortex mixed for 30 s. The resulting mixture was centrifuged, and 300 μL of supernatant was transferred to an OMEGA NANOSEP 10K size exclusion tubes, centrifuged, and transferred the supernatant into LC-MS autosampler vial.	97–109%	[26]
Rat plasma	Methyl-t-butyl ether extraction	100 μL of plasma samples were taken in 1.5 mL centrifuged tube and added 10 μL of IS and 400 μL of methyl tert-butyl ether. The resulting solution was vortex-mixed for 3 min to extract curcumin. After extraction, 300 μL of upper methyl tert-butyl ether was transferred into another tube and dried under nitrogen and reconstituted the residue into 100 μL of methanol containing 0.1% formic acid.	82.2–115.5%	[27]
mouse plasma and brain tissue	Mouse plasma and brain tissue; an addition of sodium hydroxide followed by liquid-liquid extraction	10 μL of mouse plasma (or whole brain homogenate) was spiked with 10 μL of IS and 10 μL of 0.5 M sodium hydroxide for better curcumin extraction. The solution was vortex mixed for 1 min and added 250 μL of ethyl acetate for liquid-liquid extraction. After centrifugation, the organic layer was separated and dried under vacuum, and the residue was reconstituted in 20 μL of acetonitrile and 0.01% formic acid (50:50).	Plasma: 67.0–88.4% Brain tissue: 45.8–74.6%	[28]
Human plasma	Ethyl acetate liquid-liquid extraction	3 mL of plasma samples were mixed with 10 μL of IS along with a phosphate buffer solution, followed by extracting with 3 mL of ethyl acetate. The upper organic layer was separated and transferred into a glass tube. The extraction procedure was repeated for two more times. All the supernatants were pooled and dried under nitrogen gas and reconstituted into 2 mL of methanol.	_	[35]
**Plant**				
Powdered rhizome	Microwave-assisted extraction	20 g of dry C. *longa* powder (with and without soaking solvent) was irradiated for a pre-defined time, followed by extraction with ethanol or acetone with different microwave power.	68.6% (w/o soaking) 90.5% (with soaking)	[13]
Powdered rhizome	Powdered Turmeric rhizomes, reflux in methanol	Powdered rhizomes were refluxed with methanol for 2 h at 70 C, followed by separation of aliquots, filtered, dried and resuspended in methanol for analysis	98.60%	[29]
Powdered rhizome	Pressurized liquid extraction	The optimized extractions were obtained with ethanol as extraction solvent at 100°C, pressure 1500 psi; extraction time 5 min and flush vol 60%.	93–105%	[32]
Powdered rhizome	Extraction using an ultrasonic bath	Powdered rhizomes of turmeric were extracted with 5 mL of 50, 80 and 100% of methanol. All the extracts were combined, centrifuged, and filtered for analysis.	_	[36]
Powdered rhizome	Dissolved acetonitrile	Turmeric powder; dissolved in 1 mg/mL in acetonitrile	_	[37]
Curry powder	Ultrasonication in acetonitrile-ethanol	Samples were mixed with acetonitrile: ethanol (1:2 *v*/*v*) in a centrifuge tube and shaken for 30 s. The mixture was ultrasonicated for 5 min. The supernatant was collected and used as a prepared sample solution for the analysis.	82.20%	[38]
Powdered rhizome	Pressurized liquid water extraction	Optimum extraction of the curcumin pressurized liquid water extraction was obtained at a temperature of 156.8 °C with an extraction time of 46.8 min using the solid-liquid ratio as 1:10 at a fixed 2 bar pressure. Temperature plays a vital role in extraction optimization.	_	[130]
**Formulation**				
Tiantai No. 1 pills- contains Curcuma Longa Rhizoma and other herbal medicines	Extraction with methanol and diluted HCl	Powdered Tiantai extracted by ultrasonication with an addition of 50 mL methanol and diluted HCl. Extracts were further diluted with methanol, filtered and used for analysis	96.85±4.2%	[31]
Tablet	Sonication	Powdered tablet; methanol extraction by sonication	99.8 ± 4.4%	[33]
Nanoparticles	Extracted with Dichloromethane and precipitated polymer by adding methanol	The dried nanoparticles were diluted in 1 mL dichloromethane and followed by the polymer precipitation by adding 1 mL methanol. The solution was filtered, from which 1 mL of solution taken and diluted in dichloromethane: methanol 1:1 (*v*/*v*) to adjust the absorbance	77–98%	[39]
Powdered rhizome in an herbal mix	3 stage extraction using water at high temperatures	Raw herb premix was extracted in 3 stages. In the 1st stage of extraction, the raw material was mixed with water and kept for 3 h at 80–90 °C. It was then concentrated using a rotary vacuum dryer. The resulting solid was pulverized and sieved. The 2nd and 3rd stage of extractions were carried out using water. The resulting mixture was kept for 2 h at 70–80 °C, then the solution was concentrated using a rotary vacuum dryer. The final extract was prepared by mixing 1 part of the first extraction solid and 0.5 part of each 2nd and 3rd extraction solids extraction in equal proportion.	_	[131]

**Table 3:** A summary of recent literature on simple spectroscopic and chromatographic methods used for the assay of turmeric samples.

Analytical Method	Research Objectives/Title	Matrix and Sample Preparation Method	Result	Ref
Magnetic molecularly imprinted technique and UV-Vis	A simple and rapid method for monitoring curcumin in food samples using a magnetic molecularly imprinted technique combined with UV–Vis.	Magnetic molecularly imprinted polymers of curcumin, trihydroxymethylpropyl trimethylacrylate, and polyvinylpyrrolidone	The recovery was between 79% and 89% with the limits of detection and quantification of 1.31 and 4.38 µg/mL, respectively.	[38]
UV-Vis	To develop Ultraviolet-visible spectroscopy validated method for the quantitative determination of curcumin encapsulated in poly (L-lactic acid) nanoparticles	Curcumin encapsulated in poly (L-lactic acid) nanoparticles	A UV-Vis spectroscopy method was developed to determine the concentration of curcumin on biodegradable nanoparticles and validated for linearity and inter-day, intra-day, inter-laboratory and inter-analyst precision, the limit of detection and quantification were determined.	[39]
FT-IR	To evaluate adulteration in turmeric	Turmeric powder	FT-IR was able to detect metanil yellow at 5% concentration, and FT-Raman was able to detect at 1% concentration	[41]
FT-Raman	To detect Sudan-I or metanil yellow in curry powder	Organic curry powders	The results indicated that the 1064 nm dispersive Raman system could be potentially used as a non-destructive tool for detection of chemical contaminants in a complex food matrix.	[42]
FT-NIR	Simultaneous quantification of curcuminoids	Turmeric powder	Partial least square regression (PLS-R) results showed a strong correlation with HPLC analysis	[43]
FT-NIR	To evaluate curcuminoids in turmeric powder using NIR	Crude turmeric samples	The results showed a high correlation coefficient (R^2^ > 0.93) and low standard error of cross-validation.	[44]
FT-IR, FT-Raman, and X-ray Diffraction	To compare different methods of curcumin complexation with β-cyclodextrin and to evaluate the formation of the complexes	Curcumin β-cyclodextrin complex	This complex exhibited better color stability to pH, temperature than pure colorant and had great sensorial acceptance	[45]
^1^H NMR	A rapid, accurate, and sensitive ^1^H NMR method for the quantitation of curcumin isolated from turmeric extract and compare the results with a validated with LC-MS/MS method	Curcumin samples were dissolved in DMSO-*d*6 and added 1,3,5-trimethoxy benzene was added as an internal standard	The correlation coefficients 0.998 for ^1^H NMR was and 0.995 for LC-MS/MS method in the calibration range. The measurement uncertainty for curcumin via ^1^H NMR was 5.80% as compared to 7.38% by LC-MS/MS method.	[46]
HPTLC	To develop simple and precise HPTLC methods for the simultaneous estimation of two anti-inflammatory drugs (curcumin and galangin)	Polyherbal capsule	The HPTLC method was developed and validated for linearity, accuracy, precision, detection and quantitation limits, robustness and specificity were also determined. The LOD for CUR was 18.31 ng/spot.	[47]
HPTLC	A simple and sensitive HPTLC method to develop for the simultaneous determination of salicin, curcumin, and gallic acid in herbal pain relief tablet.	Powdered tablet	The Rf values were determined as 0.16, 0.71 and 0.61 respectively for salicin, curcumin and gallic acid with the linearity ranges; salicin: 2.0–16.0μg; curcumin: 0.20–2.5 μg; gallic acid: 0.3–3.0 μg	[33]
HPLC	Isolation and identification of curcuminoids from the mother liquor by reporting the HPLC separation conditions	Commercial samples of turmeric	HPLC analysis was achieved for separation of curcumin, demethoxycurcumin, and bisdemethoxycurcumin on a C18 column using three solvents, methanol, 2% AcOH, and acetonitrile, with detection at 425 nm.	[40]
UV, FT-IR, 1H- NMR, and HPLC analysis	UV, FT-IR, 1H NMR, and HPLC were applied to construct a metabolic fingerprint to evaluate Turmeric quality.	Powdered samples were initially extracted with hexane, centrifuged, and discarded the supernatant. The residue was extracted again with methanol.	PCA analysis of the score plot of UV and HPLC analysis showed the same discriminatory patterns based on the curcuminoids content. FT-IR failed to discriminate between the same species, and ^1^H-NMR showed variability between samples in the oils/fatty acids region.	[48]
HPLC	To address the modifications suggested by the ERP for turmeric method optimization by using factorial studies to guide the optimization.	Raw materials and finished products containing turmeric roots	A single-laboratory validated method per the AOAC International guidelines was developed for curcuminoids quantification. Column temperature and extraction solvent were determined as the two most significant factors impacting the quantitation of curcuminoids. Optimum extraction was achieved with 100% MeOH, and the best separation was achieved at 55 °C column temperature.	[49]
HPLC	Developed and validated an HPLC based analytical methodology for simultaneous determination of acyclovir and curcumin within microparticles.	Samples of microparticles were extracted with 20:80 of DMSO and acetonitrile	The linear range for curcumin was determined as 0.5–20 µg/mL. Detection and quantification limits for curcumin were 91.61 ng. mL^−1^ and 128.71 ng/mL with almost complete recovery.	[134]
HPLC	Quantification of curcuminoids in commercial turmeric products, Ayurvedic medicines, and nanovesicular systems.	Powdered samples were extracted with methanol	The inter and the intraday relative standard deviation was < 2% and with almost complete 100% recovery. Limit of detection and quantification were determined to be 7.4 and 24.7 ng/mL.	[135]

**Table 4:** A brief summary of recent literature on the LC-MS analytical methods used for the assay of turmeric samples.

Purpose of Quantification/Title	Matrix and Sample Preparation Method	Instrumentation and Separation Column	MS Detection Instrumentation	LOD (ng/mL)	LLOQ (ng/mL)	Ref
Pharmacokinetic studies of curcumin in a natural turmeric matrix with two other curcumin formulations	Human plasma; Ethyl acetate extraction	Acquity High Performance LC (Waters Corporation); AQUITY BEH C18 column (2.1 × 50 mm, 1.7 μm)	Waters Xevo TQD; +ESI	1	10	[14]
Determination of curcumin and its metabolites simultaneously in phase II clinical trial	Human plasma; methanol protein precipitation	Shimadzu Prominence UFLC; Waters XTerra MS C18 column (2.1 mm × 50 mm, 3.5 μm)	AB Sciex API 3200 turbo-ion-spray triple quadrupole tandem mass spectrometer; -ESI		2.5	[25]
Determination of curcumin in rat plasma for pharmacokinetics	Rat plasma; Acetonitrile protein precipitation coupled with size exclusion chromatography	Thermo Finnigan Surveyor HPLC system; Agilent Zorbax Eclipse XDB C18 column (3.5 μm, 4.6 × 50 mm)	Thermo Finnigan LTQ mass spectrometer (ITMS/MS/MS) +ESI	0.1	1.0	[26]
Quantification of curcumin in vivo	Rat plasma; methyl-t-butyl ether extraction	Thermo Accela pump using an Agilent Poroshell SB-C18 (4.6m m× 150m m, 2.7 μm) column	Thermo TSQ Quantum; +ESI	0.5	1	[27]
simultaneous determination of curcumin in mouse plasma and brain tissue	Mouse plasma; sodium hydroxide was added to plasma, followed by liquid-liquid extraction with ethyl acetate. Brain tissue; to brain homogenate sodium hydroxide was added, followed by liquid-liquid extraction with ethyl acetate	Agilent LC 1100; an analytical Sepax BR-C18 (5 µm, 120 Å 1.0 × 100 mm) column	Agilent 6490 triple quadrupole MS; +ESI		2.5	[28]
To determine curcuminoids in *Curcuma longa* Linn.	Powdered rhizomes; refluxed in methanol	Waters ACQUITY UPLC; BEH C8 column (100.0 mm × 2.1 mm; 1.7 µm)	Waters Synapt Q-TOF Premier; -ve ionization	0.32	1	[29]
Simultaneous quantification of free curcuminoids and their metabolites in equine plasma	Equine plasma; acetonitrile protein precipitation	Shimadzu Prominence UFLC system; Waters XBridge BEH C18 column, 100 mm × 2.1 mm i.d., 2.5 um	AB Sciex QTRAP 4500 tandem mass spectrometer; ESI		0.5	[30]
Simultaneous determination of 21 bioactive components in herbal medicine and rat plasma	Medicine; 2% HCl (v/v) and methanol extraction. Rat plasma; Added 2% HCl, followed by methanol extraction	Agilent 1290 UPLC; Agilent Zorbax Eclipse Plus C18 column (4.6 mm × 150 mm, 3.5 μm)	Agilent 6410 triple quadrupole mass spectrometer	0.3	1.3	[31]
Method development and validation to study the pharmacokinetics of curcuminoids and curcumin metabolites in human blood plasma after an oral administration of bioavailable curcumin—Cureit™	Human plasma- ethyl acetate liquid-liquid extractions	Waters ACQUITY UPLC I; ACQUITY UPLC BEH C-18 (2.1 × 50 mm; 1.7 µm) column	Waters Xevo G2S Q-TOF; -ESI		1	[35]
Identification and characterization of curcuminoids in turmeric	Powdered rhizomes of turmeric were extracted with 50, 80 and 100% of methanol	Shimadzu UHPLC; Welch Ultimate UHPLC C18 column (100mm × 2.1mm, 1.8 µm)	AB Sciex Triple TOF 5600; ESI			[36]
Quantitating curcumin in food condiments and dietary supplements	turmeric powder, curry powder, and yellow mustard; 1 mg/mL in acetonitrile		MALDI-TOF-MS system (model Autoflex III Smartbeam) equipped with a 355 nm Nd: YAG laser from Bruker Daltonics (Billerica, MA, USA);		1000	[37]
Analysis of different innovative formulations of curcumin to assess relative oral bioavailability in human subjects	Human plasma; Spiked with 100 μL solution containing 1000 U of β-glucuronidase/sulfatase (EC 3.2.1.31) from Helix pomatia (Sigma, St. Louis, MO) in 0.1 M phosphate buffer (pH 6.86) and 50 μL of methanol to liberate free curcumin	Agilent 1290 HPLC; Phenomenex Kinetex XB-C18 100 Å column (2.1 × 50 mm, 2.6 micron) attached to a security guard ultra, C18, 2.1 mm pre-column	HPLC-MSMS consisted of an Agilent 1290 HPLC system with an Agilent 6460 tandem mass spectrometer; +ESI		0.5	[93]
Determine the bioavailability of curcumin in the central nervous system of mice after oral delivery of nano-curcumin	Mice plasma, brain, and spinal cord; liquid-liquid extraction with ethyl acetate	Shimadzu HPLC system; Thermo Scientific Hypersil Gold C18 column (2.1 50 mm, 5 um).	Agilent, 6410 triple quad mass spectrometer; -APCI		0.12	[96]
Identify and quantify mangiferin, berberine, kaempferol, and curcumin in a polyherbal formulation	A three-stage extraction as described in Table 2	Shimadzu HPLC (LC-2010 CHT); Chromolith high resolution RP-18 end-capped; 50 × 4.6 mm column	Agilent 6410B triple quad LC-MS/MS; +ESI	3	12.2	[131]
Quantitative analysis of curcumin and other metabolites in plant tissues	Extraction with 80:20 methanol and water with 2% formic acid	Agilent 1260 UHPLC; Agilent ZORBAX StableBond 80 Å C18 (4.6 mm × 50 mm, 3.5 μm) column	Agilent 6470 triple quadrupole mass spectrometer; −ESI	0.004	0.01	[133]
Separation, characterization, and quantitation along with the comparative pharmacological effect of curcuminoids in cerebral ischemia	turmeric powder; soxhlet extraction with petroleum ether	Water ACQUITY UPLC; Waters ACQUITY UPLC column BEH C-18 (2.1 mm × 100 mm; 1.7 μm)	Waters Synapt mass spectrometry (Synapt MS), −ESI		1	[136]
Detection of curcuminoids in human plasma	Human plasma was treated with 9:1 acetone/formic acid followed by treating with 0.1% acetic acid water/acetonitrile	Waters Alliance 2695; HyPurity C18 (2.1 × 150 mm, 3 μm) column connected to a HyPurity C18 (2.1 × 10 mm, 3 μm) guard cartridge	Micromass Quattro Platinum tandem quadrupole mass spectrometer; −ESI	0.125 nM	1 nM	[137]
Simultaneous determination of curcumin diethyl disuccinate and its active metabolite curcumin in rat plasma	Rat plasma; Acetonitrile protein precipitation	Eskpert UltraLC 100 system; Halo C8 column (4.6 × 50 mm, 2.7 um)	AB SCIEX QTRAP 6500; +ESI		1	[138]
Estimation of Curcumin in rat brain homogenate and plasma	rat brain homogenate and plasma	Waters ACQUITY UPLC; BEH C18 column (2.1 mm × 100 mm; 1.7 µm)	Waters Synapt (UHPLC/ESI-QTOF-MS/MS)	0.017	0.054	[139]
Simultaneous quantification of curcumin in rat plasma after intravenous administration	rat plasma; protein precipitation with methanol	Shimadzu HPLC; Waters ACQUITY UPLC BEH C18 (100 mm × 2.1 mm, 1.7 um,	AB SCIEX QTRAP^®^ 5500 tandem mass spectrometer; +ESI		5	[140]
to quantify curcuminoids and metabolites simultaneously in human plasma.	Human plasma; samples diluted with PBS buffer followed by ethyl acetate liquid-liquid extraction	Shimadzu HPLC; BetaBasic-8 column (2.1 mm × 50 mm, 5 µm, Thermo Hypersil-Keystone) coupled with a BetaBasic-8 guard column (2.1 mm × 10 mm, 5 µm, Thermo Hypersil-Keystone) coupled with a guard column.	AB Sciex API-3000 mass spectrometer, −ESI	1	2	[141]
simultaneous identification and quantification of three Curcuminoids in a medicinal herb.	Medicinal herb; PLE- the sample was mixed with diatomaceous earth and loaded with 15 mL of methanol into the cell. A 10-min heating process was performed, with the temperature increased up to 100 °C and maintained at a pressure of 1500 psi	Agilent 1200 Series LC system; Waters XBridge TM C18 column (150 mm × 2.1 mm i.d., 3.5 µm)	AB Sciex API 4000 triple quad MS with turbo ion spray; +ESI	0.03	0.08	[142]
to quantify curcumin in rat plasma	Sodium hydroxide was added to rat plasma sample followed by liquid-liquid extraction with ethyl acetate	Waters Alliance; Phenomenex Luna C18(2) 100A column (250 mm × 4.6 mm, 5 µm).	Quattro Micro API–Waters hexapole mass spectrometer; +ESI		0.5	[143]
To investigate the metabolism and pharmacokinetics of curcuminoids in mice tumor	plasma sample and tumor homogenate samples were extracted with acetonitrile	Finnigan Surveyor LC; Agilent Zorbax SB-C18 column (150 mm × 2.1 mm i.d., 3.5 µm) equipped with an Agilent Zorbax SB-C18 guard column (12.5 mm × 4.6 mm i.d., 5 µm).	Finnigan TSQ Quantum triple quadrupole mass spectrometer; −ESI	0.02	2	[144]

## Abbreviations

The frequently used abbreviations:

BMC	bisdemethoxycurcumin
CUR	Curcumin
CCMs	Curcuminoids
DMC	Demethoxycurcumin
FT	Fourier transform
HPLC	High Performance Liquid Chromatography
HPTLC	High Performance Thin Layer Chromatography
IR	Infrared
LC-MS/MS	Liquid chromatography coupled with tandem mass spectrometry
NIR	Near-infrared
TLC	Thin layer chromatography
uHPLC	Ultra-High Performance Liquid Chromatography
UV-Vis	Ultraviolet/visible
